# Diffuse mucocutaneous hyperpigmentation related to hydroxyurea

**DOI:** 10.1016/j.htct.2024.03.009

**Published:** 2024-07-22

**Authors:** Vishnu Sharma, Sidharth Mahajan, Vansh Bagrodia

**Affiliations:** a SMS Medical College, Jaipur, India; b Government Medical College, Amritsar, India

## Case presentation

A 63-year-old woman developed diffuse mucocutaneous hyperpigmentation post-hydroxyurea treatment for myelofibrosis. Hyperpigmentation appeared on eyelids, nasal ala, tongue, and palms without discomfort. Extensive investigations ruled out common causes; the patient had no prior dermatological conditions. Hydroxyurea was deemed causative due to temporal correlation. Counselling emphasized benign nature, recommending continued therapy with monitoring.

**Figure 1:**
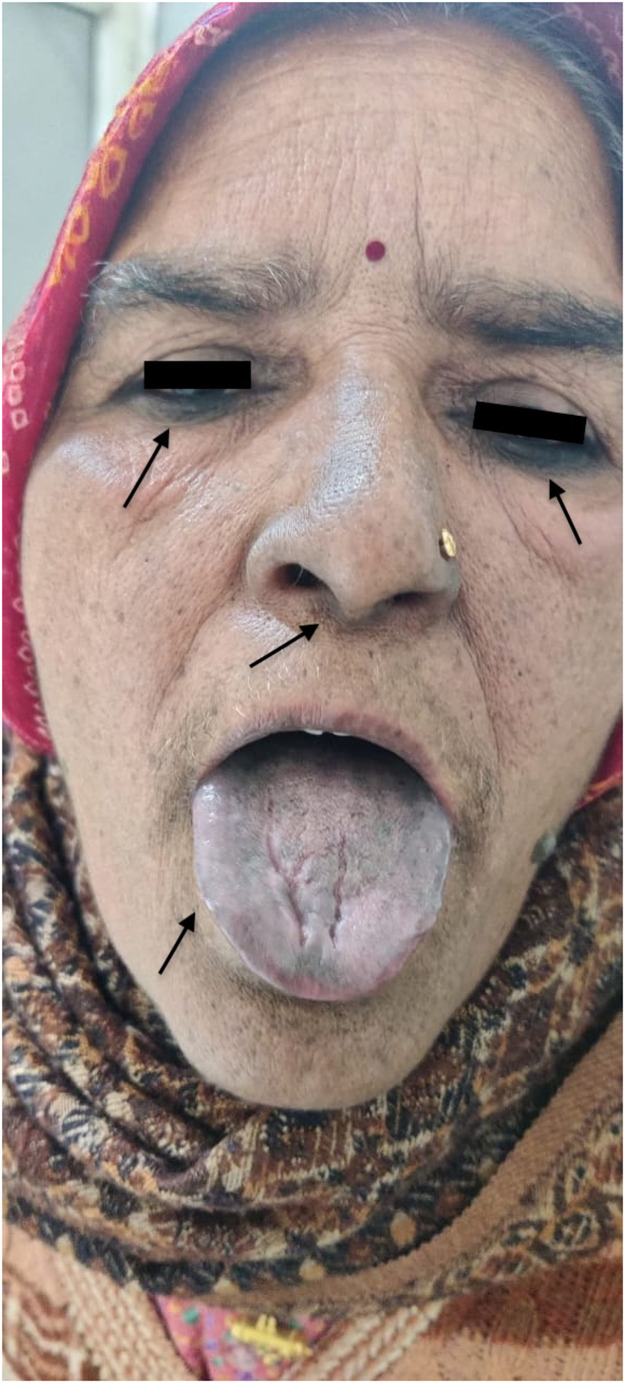
Bilateral periorbital, nasal ala, and lingual pigmentation.

**Figure 2:**
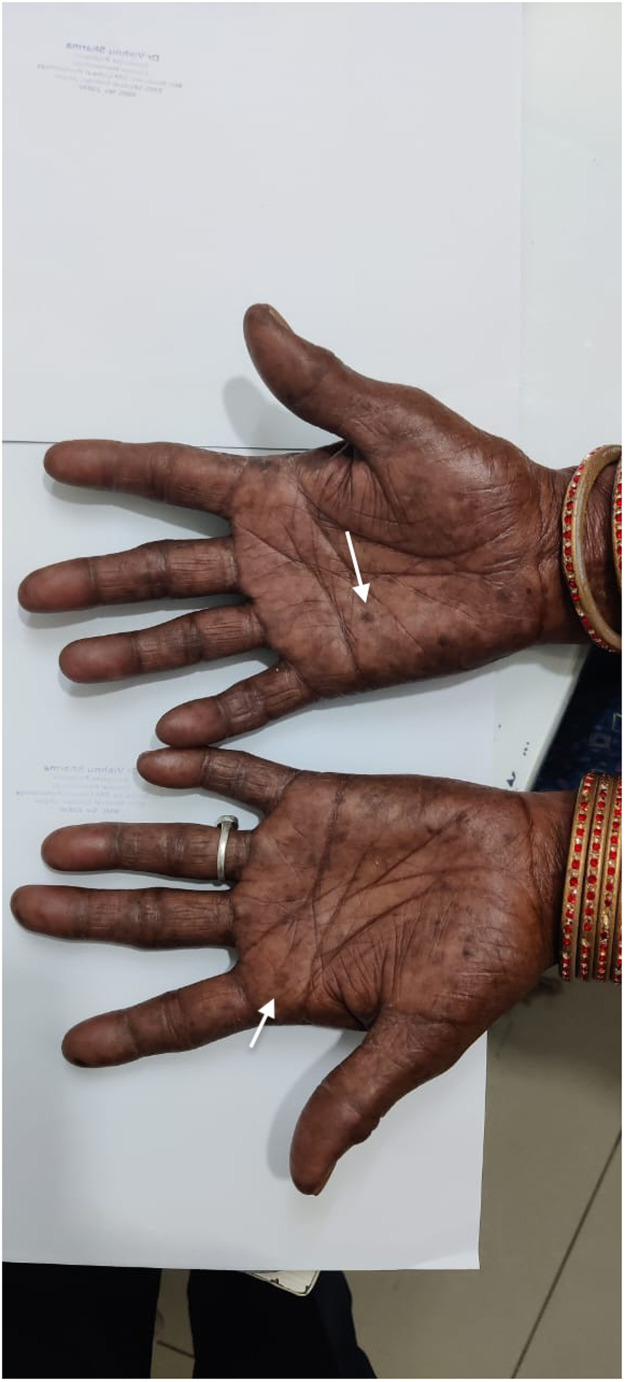
Diffuse macular palmar pigmentation.

Hydroxyurea-induced skin changes include ulcerations, melanonychia, and hyperpigmentation.^1^ Mechanism involves photosensitization, toxicity, genetic factors, possibly increased melanin, and iron deposition.^2^, ^3^, ^4^ Management includes reassurance, differential evaluation, and discontinuation in severe cases.^5^ This case differs from typical singular-site presentations, presenting a rare, multifocal pattern.^1^^,^^3^, ^4^, ^5^, ^6^ Figure 1, Figure 2.

## Conflicts of interest

The authors declare that they have no known competing financial interests or personal relationships that could have appeared to influence the work reported in this paper.
